# Substrate-induced Band Gap Renormalization in Semiconducting Carbon Nanotubes

**DOI:** 10.1038/srep03609

**Published:** 2014-01-09

**Authors:** Nicholas A. Lanzillo, Neerav Kharche, Saroj K. Nayak

**Affiliations:** 1 Department of Physics, Applied Physics, and Astronomy, Rensselaer Polytechnic Institute, 110 8^th^ Street, Troy, NY 12180; 2 Computational Center for Nanotechnology Innovation, Troy, NY 12180, USA; 3 School of Basic Sciences, Indian Institute of Technology, Bhubaneswar, India 751007

## Abstract

The quasiparticle band gaps of semiconducting carbon nanotubes (CNTs) supported on a weakly-interacting hexagonal boron nitride (h-BN) substrate are computed using density functional theory and the GW Approximation. We find that the direct band gaps of the (7,0), (8,0) and (10,0) carbon nanotubes are renormalized to smaller values in the presence of the dielectric h-BN substrate. The decrease in the band gap is the result of a polarization-induced screening effect, which alters the correlation energy of the frontier CNT orbitals and stabilizes valence band maximum and conduction band minimum. The value of the band gap renormalization is on the order of 0.25 to 0.5 eV in each case. Accounting for polarization-induced band gap changes is crucial in comparing computed values with experiment, since nanotubes are almost always grown on substrates.

Since their discovery^1^, carbon nanotubes and their unique electronic properties have been an area of great research interest. Depending on the chirality, carbon nanotubes can be classified as either metallic or semiconducting^2^,^3^,^4^. In the case of semiconducting carbon nanotubes, tight-binding methods^5^ and 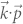 theory^6^ predict that the magnitude of the band gap is inversely proportional to the nanotube diameter. While this so-called “1/d rule” offers the possibility of fine-tuning electronic properties of carbon nanotubes by selecting the diameter, environmental polarization effects can renormalize the quasiparticle band gap and offer another route for controlling the electronic properties of nanotubes. Band gap renormalization is the reduction of the fundamental band gap due to many-body effects, which in this case is due to electron correlation.

Experimentally, it has been shown that the optical excitations of CNTs are renormalized to lower energies when the tube is covered by *N*_2_ molecules or when touched by another CNT^7^,^8^. Both of these effects were determined to be due to the polarization of the adsorbed system^9^. In the presence of a graphite substrate, it has been shown that the polarizability renormalizes the HOMO-LUMO gap of an adsorbed benzene molecule^10^. The same effect has been shown to reduce the quasiparticle band gap of graphone supported on an h-BN substrate^11^. Here, we show that this effect extends to one-dimensional semiconducting carbon nanotubes; a substrate-inducted polarization effect renormalizes the quasiparticle energy bands of the nanotube supported on a weakly-interacting hexagonal boron nitride (h-BN) substrate. The magnitude of the band gap reduction is on the order of 0.25 to 0.5 eV for the nanotubes considered. This band gap reduction is significant and must be taken into account comparing theoretical values with experimentally measured band gaps of nanotubes grown on substrates.

## Results

We have selected three semiconducting carbon nanotubes for this work with chiralities of (n,0), where n = 7,8,10. The motivation for selecting these nanotubes comes from the fact that the diameter in each case is less than 1 nm wide, which makes the GW calculations computationally tractable. Also, the band gaps in each case are larger than 1.0 eV, which makes any renormalization more effects readily apparent. The (8,0) CNT has been treated before using first-principles methods as well as the GW Approximation^20^,^21^, and offers a convenient means for benchmarking our results.

**Figure 1:**
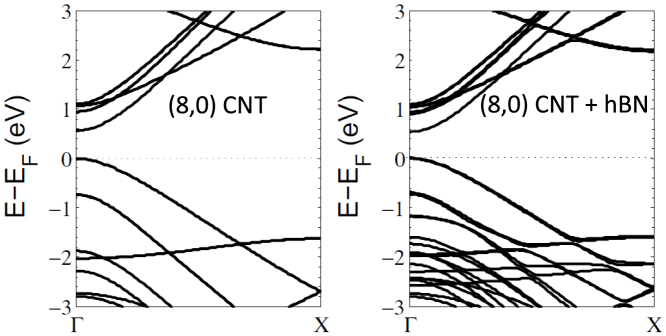
The electronic band structure for semiconducting (8,0) carbon nanotube, both free-standing and weakly-supported on an h-BN substrate.

We have first calculated the electronic band structure according to the local density approximation (LDA) for the isolated (8,0) nanotube, as well as the (8,0) nanotube supported on a monolayer of h-BN. Following the LDA calculations, we perform GW calculations to obtain the quasiparticle band gap both with and without the substrate. The results for the LDA band structure and GW-corrected band gap at the Γ-point of the (8,0) nanotube are shown in Figure 1.

While the qualitative features of these band structure plots are correct, the value of the direct band gap is severely underestimated by the LDA. In the (8,0) CNT, the GW corrections open up the gap from 0.56 eV (at the LDA level) to 1.80 eV (GW) at the Γ point. The value of our GW-corrected band gap for the (8,0) CNT agrees to within 0.07 eV of previous studies^20^,^21^. The presence of the dielectric h-BN substrate leaves the LDA band gap essentially unchanged. However, the long-range screening effects present in the GW calculation, due to the dielectric substrate, show that the band gap is reduced to around 1.45 eV in the presence of the substrate; a renormalization of approximately 0.25 eV.

**Figure 2:**
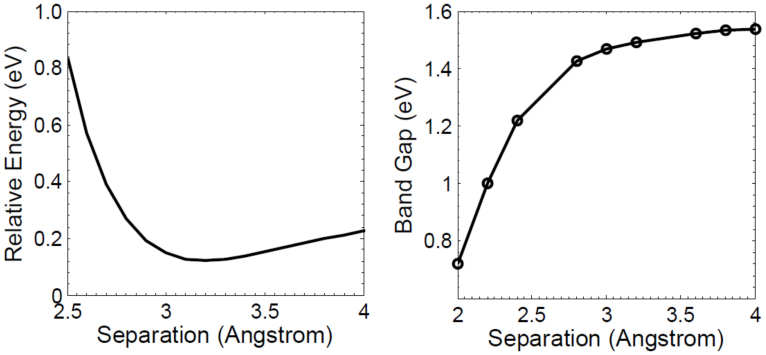
The total relative energy and electronic band gap as functions of the CNT/hBN separation.

To investigate the effect of the altering the CNT/hBN separation, we have calculated the total relative energy as well as the renormalized band gap as functions of separation. The relative energy is the total energy of the CNT-hBN system at a given separation minus the total energy of the CNT-hBN system at the equilibrium separation (3.15 Angstrom). These are plotted in Figure 2.

We see that the CNT/hBN configuration has a minimum energy configuration in the 3.1 to 3.2 Å separation range, which is similar to the minimum energy range of graphene and graphone on hBN^11^. While the LDA does not take into account the Van der Waals interaction, it includes a fortuitous cancellation of errors and gives reasonable values of lattice constants for weakly bound layered materials. The fact that the electronic band gap exhibits a stronger renormalization for smaller tube-substrate separations is indicative of the increased screening as the CNT is brought closer to the substrate.

**Figure 3:**
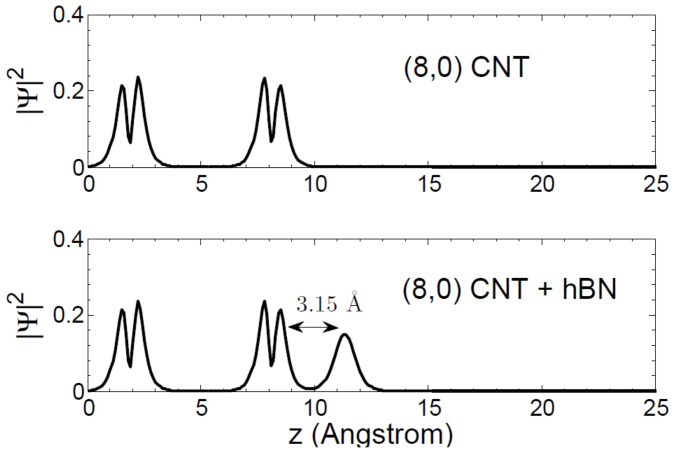
The electronic density along the z-direction for the isolated (8,0) carbon nanotube and the (8,0) carbon nanotube supported on an h-BN substrate. There is negligible density overlap between the CNT and the substrate.

To ensure that the effects observed are due to dielectric screening and not a covalent or charging interaction between the nanotube and the substrate, we have analyzed the total electronic density cut along the z-direction, which is perpendicular to the substrate. This is plotted in Figure 3.

For the isolated (8,0) carbon nanotube, the two separate peaks in the density correspond to the opposite edges of the nanotube diameter, while the single-atom thick hBN layer gives rise to a lone peak in the density along the z-axis. As evident in Figure 3, there is negligible density overlap between the nanotube and the substrate, indicating that any renormalization effects are due to a long-range screening interaction as opposed to any short-range covalent interactions.

Following the analysis of the (8,0) nanotube on the substrate, we then performed the same analysis for the (7,0) and (10,0) nanotubes. The (7,0) and (10,0) nanotubes are slightly smaller and slightly larger in diameter, respectively, than the (8,0) nanotube. We first calculate the LDA band structure both with and without the substrate and then calculate the GW corrections to the band gap in each case. Our results for all three semiconducting nanotubes are summarized in Table I.

For the (7,0) CNT we see the quasiparticle band gap reduces from 1.82 eV when isolated to 1.48 eV when supported on h-BN, and for the (10,0) CNT we see the quasiparticle band gap reduce from 1.89 eV when isolated to 1.53 eV when supported on h-BN. It is worth noting that in each semiconducting nanotube, the GW corrections were calculated at four equally spaced points in k-space between Γ and *X*, as well as the corrections at the X-point. For each nanotube considered, the band gap reduction throughout the Brillouin Zone had the same value of roughly 0.35 eV. This is reminiscent of the so-called “scissor-shift” in carbon nanotubes, in which the GW Approximation raises the energy bands with respect to the LDA values by a more-or-less constant value. It is interesting to note that for the semiconducting nanotubes considered with diameters ranging from 5.48 Å to 7.83 Å, there is no significant diameter dependence of the band gap renormalization. However, with this small relatively sample size we cannot say with certainty whether or not this is true for larger nanotubes.

## Discussion

At the DFT level, the only effect of adding the h-BN substrate in the LDA is the appearance of addition bands (belonging to h-BN) well below and above the Fermi level. The addition of the h-BN substrate has a negligible effect on the LDA band gaps of the carbon nanotubes, leaving the values essentially unchanged in each case. This is similar to the results seen for placing graphene/graphone on an h-BN substrate^11^, in which the LDA fails to take into account non-local screening but the GW Approximation shows significant band gap renormalization due to the polarization of the substrate.

The band gap reduction in each case is the result of the polarization-induced screening effect of the substrate. The physical cause of this effect is identical to that discussed in previous studies of graphone deposited on h-BN^11^, a benzene ring deposited on graphite^10^, and graphene nanoribbons (GNRs) deposited on substrates^24^,^25^,^26^. Namely, the presence of a polarizable material (dielectric h-BN) reduces the dynamically screened Coulomb interaction (*W*) between the nanotube and the substrate. The screened interaction is related to the bare interaction (*V*) via 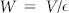, so it is clear that a larger dielectric constant will result in a smaller screened Coulomb interaction. Since the screened interaction is reduced, the corresponding self-energy matrix (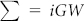) will be reduced and likewise the contribution to the quasiparticle band gap is reduced. Since the LDA calculations do not include dynamically screened Coulomb interactions, they do not capture the substrate-induced changes in the band structure. In principle, the band gap of an adsorbed material can be fine-tuned by controlling the dielectric environment of the substrate. Materials with a large dielectric constant will give rise to a larger band gap renormalization in the adsorbed material.

In summary, we have shown that quasiparticle band gaps of semiconducting (7,0) (8,0) and (10,0) carbon nanotubes supported on a weakly-interacting h-BN substrate are reduced due to a polarization-induced screening effect. The screening effect is significant, reducing the band gap by roughly 0.35 eV in each case. This extends the scope of previous studies which have looked at the same effect on benzene and graphene supported on substrates and opens the door for future studies of band gap modulation in carbon-based structures at the nanoscale. Future work will include studying the band gap renormalization in larger nanotubes with diameters larger than 1.0 nm to allow closer comparison with experiment, as well as investigating the extent to which screening modifies the quasiparticle states away from the band gap in a CNT deposited on a substrate.

## Methods

Our calculations were performed in the ABINIT^12^ software package, which is a planewave pseudopotential implementation of density functional theory^13^,^14^. We have used Troullier-Martins pseudopotentials^15^ for C, B and N atoms with a plane-wave cutoff energy of 30.0 Hartree. Calculations were performed in the local density approximation (LDA)^14^. Our unit cells for the nanotubes have dimensions of 22.14 Å along the x-direction, 4.26 Å along the y-direction, and 25.0 Å along the z-direction. The CNTs are oriented to run along the y-axis, and the h-BN substrate is periodic in the x-y plane. The h-BN sheet extends 22.14 Å in the direction perpendicular to the nanotube axis. Along the z-direction, we chose a separation of 3.15 Å between the bottom of the CNT and the h-BN layer, along with more than 10 Å of vacuum separating the CNT from the periodic image of the next h-BN layer. The separation of 3.15 Å was chosen since this is the optimized separation in the local density approximation for graphene on h-BN. Due to the weak interaction between carbon and h-BN, there is a shallow potential energy minimum around 3.15 Å, and the energy differences for separations in the range of 3.0 to 3.5 Å differ by less than 0.1 eV. We do not expect small changes in CNT-hBN separation to dramatically affect the polarization-induced band gap changes that we seek to identify. Our substrate is chosen to be just one-atom thick in order to efficiently simulate the presence of a dielectric while keeping the problem computationally tractable.

First-principles density functional theory (DFT) calculations are known to underestimate the band gaps of semiconductors by as much as a factor of 2. Furthermore, DFT fails to account for both dynamical electron correlation as well as static, long-range image potential effects for an electron at a metal-semiconductor interface^16^. To remedy this, we have calculated the quasiparticle band gaps using the GW Approximation^17^,^18^. After solving the Kohn-Sham equation, 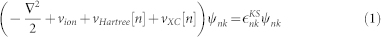
the corresponding single-particle wavefunctions are used as a first approximation for the quasiparticle wavefunctions. The GW corrections are treated as perturbations to the DFT wavefunctions. The quasiparticle energies (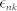) are then found by solving the Dyson equation: 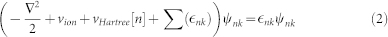
The self-energy matrix, 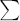 is approximated as the one-particle Green's Function (G) times the dynamically screened Coulomb interaction (W): 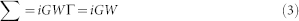
while the vertex function, Γ is approximated as a local and instantaneous function.

For both LDA and GW calculations we use a Γ-centered 2 × 18 × 1 Monkhorst-Pack k-point grid. We have checked that additional sampling along the periodic direction of the h-BN layer does not change the results in a significant way since the length of this dimension is on the order of 20 Å. For the GW calculations, we find converged results to within 0.1 eV using cutoff energies of 8 Rydberg for the wavefunctions used to compute the dielectric function and the self-energy matrices. We use 200 bands for the calculation of both the dielectric function and the self-energy matrix elements. We have also used a one-dimensional Coulomb cutoff for the isolated CNTs and a two-dimensional Coulomb cutoff for the substrate-supported CNTs in order to achieve proper convergence with respect to the vacuum layer^19^. These cutoffs truncate the Coulomb interaction beyond a specified cutoff radius to avoid complications due to small but non-zero Coulomb forces at the edges of the computational box, and are necessary in order to achieve convergence with respect to the number of k-points used^22^. We have checked that increasing the vacuum by 10 Å changes the values of the band gap by less than 70 meV. The GW calculations for the semiconducting nanotues were carried out using the Plasmon-Pole Model.

To check that the effect of the CNT interactions with neighboring periodic images of CNTs when using a two-dimensional Coulomb Cutoff is negligible, we have calculated the band gaps of CNTs (without a substrate) using both one and two-dimensional Coulomb Cutoff techniques and find that the band gaps in each case agree to within 0.1 eV. This is to be expected, since the axes of neighboring CNTs are 22 Å away from one another and the Coulomb interaction between them is extremely small. Finally, we have checked that adding an additional layer of h-BN to the substrate changes the values of the renormalized band gaps by less than 0.1 eV, indicating that a single layer is sufficient to elucidate the effects of dielectric screening that we describe. We note that single layers of h-BN have been used in other works^23^ to model the dielectric environment of carbon-based systems and agreement with experiment was found.

## Author Contributions

N.L. wrote the main manuscript text and prepared figures 1–3 with help from N.K. and S.N. All authors (N.L., N.K. and S.N.) reviewed the manuscript.

## Figures and Tables

**Table 1:** The LDA and GW band gaps for the (7,0), (8,0) and (10,0) carbon nanotubes, with and without the h-BN substrate. All values are in eV

	LDA	GW
(7,0) CNT	0.45	1.82
(7,0) CNT + hBN	0.48	1.48
(8,0) CNT	0.56	1.80
(8,0) CNT + hBN	0.53	1.45
(10,0) CNT	0.90	1.89
(10,0) CNT + hBN	0.89	1.53
